# The Risk of Bleeding Complications in Intra-Articular Injections and Arthrocentesis in Patients on Novel Oral Anticoagulants: A Systematic Review

**DOI:** 10.7759/cureus.17755

**Published:** 2021-09-06

**Authors:** Muhammad Yasir Tarar, Xin Yin Choo, Shoaib Khan

**Affiliations:** 1 Trauma and Orthopaedics, Salford Royal NHS Foundation Trust, Manchester, GBR; 2 Trauma and Orthopaedics, Blackpool Victoria Hospital, Blackpool, GBR; 3 Trauma and Orthopaedic, St Helens and Knowsley NHS Trust, Manchester, GBR

**Keywords:** arthrocentesis, intra-articular injection, bleeding, doac, noacs

## Abstract

Novel oral anticoagulants (NOACs) are increasingly prescribed blood-thinning medication. Surpassing Warfarin, NOACs are more favored and extensively used in paroxysmal atrial fibrillation, acute coronary syndrome, and in elderly patients. Well-known benefits of novel oral anticoagulants include predictable pharmacokinetics, pharmacodynamics, and shorter half-life. However, as with any anticoagulant, there are bleeding risks with NOACs. There is a lack of evidence and consensus on the management of novel anticoagulants for intraarticular injections and arthrocentesis. This systematic review aims to analyze the risk of bleeding complications in patients on novel oral anticoagulants who underwent joint injections and arthrocentesis to help physicians in the decision-making and consenting process. A literature search of three online databases was completed using the Cochrane methodology for systematic reviews. Eligibility criteria included any study that reported bleeding complication rates in adult patients on novel oral anticoagulants that had a joint injection or aspiration whilst continuing their regular oral anticoagulation. All studies with any number of patients and published in any language were included. Review articles and systematic reviews were excluded. The search of databases resulted in a total of 310 articles. After screening, a total of four articles were deemed suitable to be included in the analysis. These described a total of 668 patients undergoing injections/aspiration procedures with patients on three different novel oral anticoagulants namely Rivaroxaban, Apixaban, and Dabigatran. Only one patient joint had a bleeding complication and the patient was on Dabigatran. The results of this systematic review show that it is relatively safe to perform joint injections and arthrocentesis whilst continuing on Novel oral anticoagulation.

## Introduction and background

Warfarin, a vitamin K antagonist, was first introduced in the 1950s to treat venous thromboembolic (VTE) diseases like stroke, atrial fibrillation, deep venous thrombosis, and pulmonary embolism. Since then, it has been the choice of anticoagulation until the early 2000s. Direct oral anticoagulants (DOACs) or novel oral anticoagulants (NOACs) were first marketed in the UK in 2008 [1]. This medication has revolutionized the way anticoagulants are prescribed. NOACs are increasingly prescribed, surpassing warfarin, and are more favored, especially in paroxysmal atrial fibrillation, acute coronary syndrome, and elderly patients [1,2]. Novel oral anticoagulants are preferred because of their predictable pharmacokinetics, pharmacodynamics, and shorter half-life. They do not require regular monitoring and dietary restriction [3,4]. However, as with every anticoagulant, they carry the risk of bleeding. A study has shown that the bleeding risk with NOACs to treat atrial fibrillation in the elderly was up to 4.4% for major bleeding and 5.7% for clinically relevant non-major bleeding [5].

It is estimated that 28.9% (17.8 million) of people have musculoskeletal conditions in the United Kingdom. A study has shown that one-third of patients presenting at general practice with musculoskeletal problems are treated with a steroid injection in the knee joint [6]. Joint injections and arthrocentesis are routinely performed for diagnostic and therapeutic purposes in various inflammatory and infective arthropathies. These procedures are often performed in the elderly population, who are on polypharmacy, including one or more anticoagulants at times. It is estimated that in England, up to 2.4% of the population aged 18 years or older are on anticoagulation therapy, with up to 1.25 million people currently prescribed oral anticoagulants [7,8]. Current recommendations are to discontinue Novel oral anticoagulants at least 24-48 hours before surgery, subject to bleeding risk [9]. There is a lack of evidence and consensus on the management of Novel anticoagulants for intraarticular injections and arthrocentesis.

## Review

Methodology

Literature Search

A literature search of MEDLINE (1946 to present), EMBASE (1974 to present), and Cochrane CENTRAL (1988 to present) databases was conducted using any combination of the keywords “joint,” “intraarticular,” “arthrocentesis” and “novel oral anticoagulant” and “direct oral anticoagulant” in February 2021 for articles published in any language with no publication year limit.

Study Selection

Any study design, including randomized controlled studies, prospective cohort studies, retrospective cohort studies, case-control studies, and case series which included more than five patients were included in this study. Case reports were excluded.

Assessment of Methodological Quality

Two reviewers independently screened the titles and abstracts of all identified studies for inclusion. Duplicates were removed. Full texts of studies considered eligible were retrieved and reviewed. The reference lists of all included articles were searched for any additional articles not identified through the database search. Disagreement for inclusion was discussed between the reviewers and, if not resolved, senior author input was obtained.

Data Extraction

**Figure 1:**
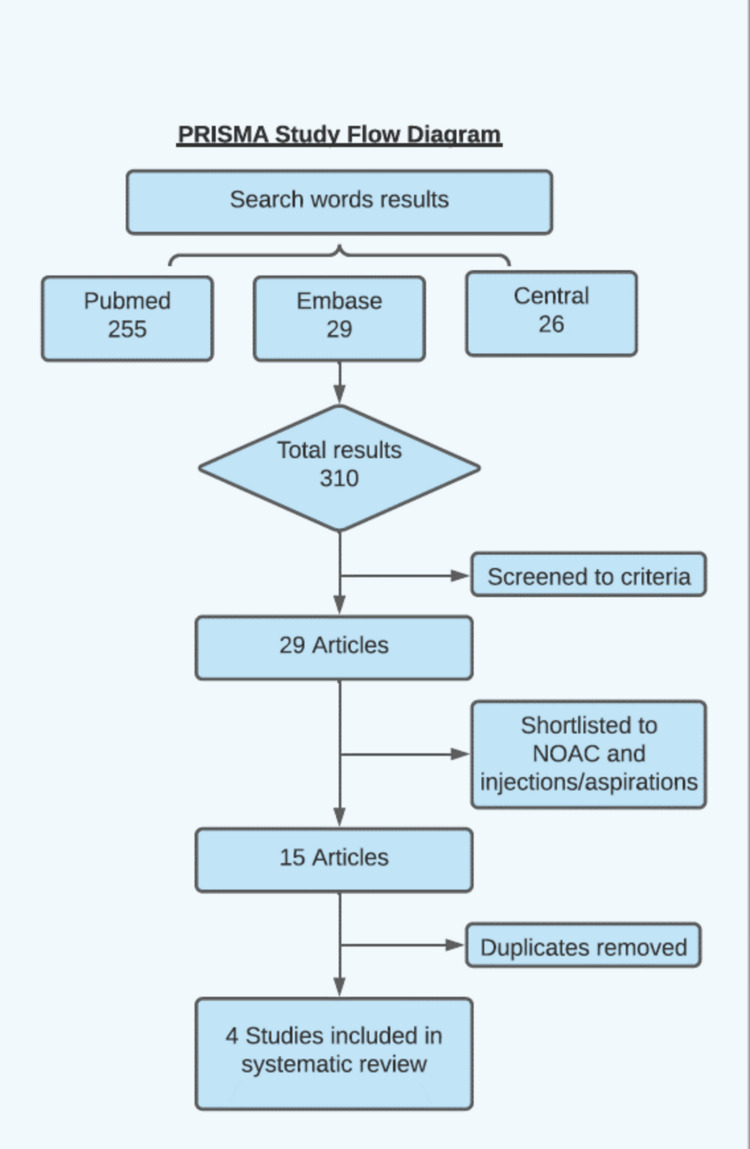
PRISMA study methodology This flow diagram shows the literature search process using PubMed, Embase, and Central which resulted in a total of 310 articles. After screening, a total of four articles were deemed suitable to be included in the analysis.

The Preferred Reporting Items for Systematic Review and Meta-Analyses (PRISMA) methodology was used. This process is summarized in Figure 1. The Cochrane Risk of Bias Tool was used to assess the risk of bias in included RCTs. The Methodological Index for Nonrandomised Studies (MINORS criteria) tool was used to assess bias in observational studies, as shown in Table 1.

**Table 1:** MINORS assessment score The methodological index for nonrandomised studies (MINORS criteria) tool was used to assess bias in the selected studies [14]. MINORS: Methodological Index for Nonrandomised Studies.

Articles included	Yui et al. [10]	Nord et al. [11]	Guillen-Astete et al. [12]	Mian et al. [13]
Aim clearly stated	2	2	2	2
Inclusion of consecutive patients	2	2	2	2
Prospective collection of data	0	0	0	0
Endpoints appropriate to the aim of the study	2	2	2	2
Unbiased assessment of the study endpoint	0	0	0	0
Follow-up period appropriate to the aim of the study	2	0	2	2
Loss to follow up less than 5%	2	0	2	2
Prospective calculation of the study size	0	0	0	0
Total score (out of 16)	10	6	10	10

Primary Outcome

This study aimed to analyze the risk of bleeding complications in patients on DOACs/NOACs who underwent joint injections and arthrocentesis to help physicians in the decision-making and consenting process.

Results

The search of databases with keywords resulted in a total of 310 articles found. Following initial screening and following the criteria, 29 articles were selected. On further review and removal of duplications, four were selected to be included in the systematic review.

The included studies involved a total of 668 patients. Three studies have specified the number of injections performed, and that summed up to 1280 injections. All of these injections were performed in patients over age 70 years old. Two studies, Yui et al. and Nord et al. included the patients on Rivaroxaban, Apixaban, and Dabigatran [10,11]. The study conducted by Guillen-Astete and Quiñones-Torres [12] only included the patients on Dabigatran. In the studies by Yui et al. [10] and Nord et al. [11], the highest number of procedures were done in patients on Rivaroxaban, then Apixaban, and lastly, Dabigatran. In their study, none of the patients developed any bleeding complications. In the study by Guillen-Astete and Quiñones-Torres [12], 117 injections were given for 68 patients, and only one patient developed hemarthrosis and two developed increasing pain. This is summarized in Tables 2-3.

**Table 2:** Demographics and Novel oral anticoagulant details relating to the selected articles. All four studies were retrospective and studied joint injections performed in patients over the age of 70 years old. The follow-up time varied among all four studies. Only Nord et al. included information about the needle size of 25, 27G [10-13].

Author	Year	Study type	Age mean/median	Sex (M/F)	Method (fluoroscopic/USG/manual)	Anticoagulant agent, NOAC/DOAC	Follow-up time	Needle size used
									Yui et al. [10]	2017	Retrospective	75	49/51	USG guided/manual	Rivaroxabn, Dabigatran, Apixaban	0-1307 days	Not stated
Nord et al. [11]	2018	Retrospective	80.1	39/61	Not stated	Rivaroxabn, Dabigatran, Apixaban	Not stated	25,27G
Guillen-Astete and Quiñones-Torres [12]	2017	Retrospective	Mean 71	52/48	USG guided/manual	Dabigatran	15 days	Not stated
Mian et al. [13]	2019	Retrospective	77	65.7/44.3	Not stated	Not stated	1 month	Not stated

**Table 3:** Procedures and complications. The included studies summed a total of 668 patients. The study by Yui et al. had the largest sample size and was conducted retrospectively over the longest duration among the four studies which was six years. Only the study by Guillen et al. had complications [10-13].

Author	Total patients	Total injections	Knee	Shoulder	Rivaroxban	Apixaban	Dabigatran	Bleeding	Severe pain	Infection
											Yui et al. [10]	483	1050	442 injections	142 injections	548 injections	325 injections	177 injections	0	0	0
Nord et al. [11]	90	113	56 injections	27 injections	42 patients	36 patients	12 patients	0	0	0
Guillen-Astete and Quiñones-Torres ​​​​​​​[12]	68	117	68 injections	49 injections	0	0	117 injections	Hemarthrosis 1	Increasing pain 2	0
Mian et al. [13]	27	Not stated	Not stated	Not stated	Not stated	Not stated	Not stated	0	0	0

Discussion

Novel oral anticoagulants serve a vital role in providing prophylaxis and treatment of conditions like atrial fibrillation, stroke, deep venous thrombosis, and pulmonary embolism. Discontinuation of these agents for joint injection can predispose patients to a high risk of thromboembolic events [15,16]. A search via the General Practice database revealed that the average age of women and men suffering a stroke is 77 years and 71 years, respectively. Several studies have compared the use of the traditional vitamin K antagonist and DOAC and found that DOAC is a feasible option to reduce the risk of VTE events [17]. The purpose of this systematic review was to evaluate complications in patients on DOAC undergoing arthrocentesis or joint injections.

The present study demonstrated that DOAC is safe to be continued in patients undergoing arthrocentesis and joint injections. This finding is in concordance with the included studies. In England, it is estimated that one in ten adults have been diagnosed with osteoarthritis, with knee joint being the commonest [18-20]. A study has shown that around one in five patients presenting to General Practice in the UK complains of musculoskeletal issues in which a significant number of patients have knee problems. One in three of these knee consultations undergo steroid injections [6]. Arthrocentesis and joint injections are both commonly performed procedures worldwide. Conventionally, these procedures are performed by delineating the surface anatomy of joints which guides needle insertion. Several studies have compared landmark and ultrasound-guided arthrocentesis. They found that the ultrasound-guided method is easier and quicker to perform as compared to the landmarked approach [21-23]. Nonetheless, the introduction of a needle carries a risk of damage to surrounding ligaments, blood vessels, and nerves.

Theoretically, patients on oral anticoagulants are at a higher risk of bleeding complications during surgical procedures due to inhibition of factor IIa or Xa [24]. Apixaban, Rivaroxaban, and Edoxaban act by inhibiting factor Xa directly. Dabigatran binds to thrombin (factor IIa) directly to inhibit its action [25]. For major invasive procedures, NOAC/DOAC is discontinued. Currently, there are no guidelines available on the management of NOAC/DOAC in arthrocentesis or joint injections. Therefore, we conducted this systematic review to evaluate complications related to joint injections or arthrocentesis in patients on NOAC/DOAC to provide evidence in developing the guideline.

Our study evaluates the bleeding complication in patients with a joint injection or arthrocentesis whilst on anticoagulants, specifically DOACs. After a thorough search, four articles that fit our inclusion criteria were evaluated. Out of all four studies, only one study had a joint injection-related complication.

Guillen-Astete and Quiñones-Torres reported complications of joint injections in 68 patients on Dabigatran. In their report, 11 (16.1%) of patients presented within 15 days of the procedure with increasing or severe pain, in which one suffered from haemarthrosis. The patient was treated conservatively. It was unclear whether this patient had an ultrasound-guided procedure [12].

Nord et al. on patients anticoagulated with therapeutic DOAC doses found no post-injection complications [11]. Similarly, Main et al. reported no immediate or late complications in patients receiving arthrocentesis while on therapeutic doses of DOAC [13]. Yui et al. evaluated complications related to joint injections in patients on DOAC, on a combination of DOAC and aspirin and DOAC and Clopidogrel [10]. They found no complications post-injection. Although, the evidence reported increased bleeding risk with combined antiplatelet and anticoagulation treatment [26]. None of the studies reported the duration of anticoagulation before injections, which appears to impact bleeding risk [27].

Yui et al. and Nord et al. studied three DOACs: Rivaroxaban, Apixaban, and Dabigatran [10,11]. Guillen-Astete et al. included patients on Dabigatran, and Mian et al. did not specify the type of DOAC studied [12,13]. Danish databases, Norwegian Patient Registry and Norwegian Prescription Database evaluated the efficacy and bleeding risk with atrial fibrillation on DOACs compared with warfarin. These databases reported a lower rate of major bleeding and intracranial bleeding with Dabigatran compared to warfarin [28,29]. This finding was also supported by a cohort study in primary care in the UK [30]. In our review, Dabigatran was related to bleeding complications post-injection in comparison to other DOACS. However, Dabigatran-associated bleeding was found in only one patient; therefore, the bleeding correlation seems to be insignificant. 

Apart from Main et al., the knee was the most common joint injected [12]. One of the most common conditions treated with joint injection is osteoarthritis. All four articles reported the patient age group to be more than 70 years by a median or mean calculation method. Nord et al. studied both groups of patients receiving and not receiving DOAC treatment and noted that the age of patients taking DOAC was higher than those not taking DOAC [11]. This can be explained because thrombosis or emboli-related phenomena are prevalent in older age groups.

Our study has several limitations. First, the data were drawn from low evidence studies which in turn conclude a systematic review of low-level evidence. Second, information about follow-up time and anticoagulation levels was limited. Only Yui et al. had published data on follow-up [10]. Third, there was also limited information on the technique of the joint injection, which can have an impact on injury to surrounding structures. The strength of the study is that it has included literature published in all languages. The literature was translated to the English language for review. From our knowledge, this is the first systematic review conducted on the subject under discussion.

## Conclusions

Currently, available data suggest that DOACs are safe to use in joint injections. Only one study had described a patient on Dabigatran; a direct thrombin inhibitor experienced hemarthrosis post-injection. However, only small populations were studied and did not cover the same anticoagulants. With DOACs being increasingly prescribed, bigger studies involving a larger population, various DOACs, and anticoagulation levels can be studied to ensure patient safety whilst on DOACs.
